# Do Cues Matter? Highly Inductive Settlement Cues Don't Ensure High Post-Settlement Survival in Sea Urchin Aquaculture

**DOI:** 10.1371/journal.pone.0028054

**Published:** 2011-12-05

**Authors:** Benjamin Mos, Kenneth L. Cowden, Shaun J. Nielsen, Symon A. Dworjanyn

**Affiliations:** 1 National Marine Science Centre, Southern Cross University, Coffs Harbour, New South Wales, Australia; 2 Centre for Marine Bio-innovation and School of Biological, Earth and Environmental Sciences, University of New South Wales, Sydney, New South Wales, Australia; Laboratoire Arago, France

## Abstract

Increasing settlement and post-settlement survival during the critical transition from planktonic larvae to benthic juveniles will increase efficiency for sea urchin aquaculture. This study investigated the effects of temperature and settlement cues on the settlement and post-settlement survival of the sea urchin *Tripneustes gratilla* during this phase. The current commercial methodology, which utilises natural biofilm settlement plates, was tested and resulted in low settlement (<2%) and poor post-settlement survival (<1% of settled urchins). In laboratory trials, settlement was high and unaffected by temperatures between 24 and 30°C, but significantly decreased at 33°C. Development of spines, however, was significantly affected by temperatures over 29°C. Mirroring this result, post-settlement survival was optimal between 24–28°C. In laboratory assays, the macroalgae *Sargassum linearifolium* and *Corallina officinalis*, and seawater conditioned with these algae, induced significantly higher settlement (>90%) than a natural biofilm (∼25%). The addition of macroalgae-conditioned seawater to natural biofilm significantly increased settlement rates (>85%). Mixed consortia and single strains of bacteria isolated from macroalgae, biofilms and adult conspecifics all induced significant settlement, but at significantly lower rates than macroalgae. No evidence was found that higher rates of settlement to bacteria on macroalgae were generated by a cofactor from the macroalgae. Age of bacterial cultures, culturing bacteria on solid and liquid media and concentration of nutrients in cultures had little effect on settlement rates. Finally, macroalgae-conditioned seawater combined with natural biofilm settlement plates induced significantly higher settlement than to the biofilm plates alone in a commercial scale trial. However, high post-settlement mortality resulted in equivalent survival between treatments after 25 days. This study highlights that settlement studies should extend to post-settlement survival, which remains poor for *T. gratilla* and is a significant obstacle to increasing efficiency for aquaculture.

## Introduction

There has been a substantial decrease in the productivity of the global sea urchin fishery [1]. Individual sea urchin fisheries have followed the familiar pattern, seen in many invertebrate fisheries, of over-exploitation and collapse, as stocks are sequentially fished further and further from main markets [1]–[4]. This decrease is driving the development of culture methods for several commercially important sea urchin species [5]–[8]. The refinement of these culture methodologies to increase efficiency will assist sea urchin aquaculture to supply the increasing demand for sea urchin product caused by the declines in wild fisheries production.

One critical phase in the culture of sea urchins is the transition from planktonic larvae to benthic juveniles. Larval settlement and metamorphosis rates can be highly variable, ranging from 0–90% [7], [9]–[15]. Similarly, high mortality within the first weeks (94% *Paracentrotus lividus* [7]; >90% *Tripneustes gratilla* [8]) reduces the number of urchins reaching the transition to feeding on macroalgae, after which mortality is typically low [16]. These problems limit the efficiency of sea urchin aquaculture as the larval phase of sea urchins is often long (e.g. 30–40 days [17]), requiring a substantial investment of resources. Increasing settlement rates and maximising post-settlement survival is a major challenge in the realisation of profitable sea urchin aquaculture.

In production, sea urchin larvae are typically induced to settle to diatom dominated biofilms cultured on vertical plates [8], [18], [19]. However, in laboratory experiments larvae respond to a wide range of cues including macroalgae [11]–[14], [20]–[22], conspecific adult urchins [11], specific chemicals [21], [23]–[28] and bacteria [13]. Bacteria have received particular attention given that macroalgae with bacteria removed induce little or no settlement [11], [13], [15]. The identification and use of highly inductive cues could reduce the variability and unpredictability often observed in settlement of invertebrates to biofilms [29]–[32]. For example, the use of highly inductive cues has been shown to increase settlement rates in abalone aquaculture [29], [33], [34]. Similarly, settlement of the sea urchin *Strongylocentrotus intermedius* can be bolstered with the growth of macroalgae, such as the brown alga *Hizikia fusiformis* or the green alga *Ulvella lens*, amongst biofilms [35].

The objective of this study was to investigate ways to increase survival in the transition from planktonic larvae to macroalgae-consuming benthic juveniles in the aquaculture of the tropical sea urchin *Tripneustes gratilla. T. gratilla* is a quick growing species [17], [36] with a high market value. It is being targeted as an aquaculture species [8], [17], [37] and is produced in small quantities for restocking in Japan [1], [8] and as food in the Philippines [37]. Its broad feeding preferences mark it as a promising species for integrated aquaculture (Seymour et al., unpublished manuscript) and it is being studied as a biological control for invasive macroalgae in the Hawaiian Islands.

To get a baseline of the effectiveness of the current commercial methodology for inducing settlement of *T. gratilla*, a commercial trial was conducted assessing settlement and post-settlement survival. Temperature was tested as a factor influencing both settlement and post-settlement survival in a laboratory experiment. To identify potential cues to enhance settlement, laboratory experiments assessed the ability of biofilms, macroalgae, seawater conditioned with macroalgae, mixed consortia and individual strains of bacteria, and supernatants of all bacteria to induce settlement. Because the physiological state of bacteria may influence the type of metabolites they produce [38], and hence influence their inductive capacity, the effects of bacterial culture method, the age of bacterial culture and the presence of macroalgae were tested in laboratory experiments. Finally, the most practical, highly inductive cue, as determined by the laboratory experiments, was tested in a commercial trial to determine the effectiveness of a highly inductive cue in increasing settlement at commercial scales, and to assess the impact on post-settlement survival.

## Materials and Methods

### Study Organisms

Adult *Tripneustes gratilla* broodstock were collected (New South Wales Department of Industry and Investment, permit P10/0023-1.0) near Coffs Harbour, New South Wales (30°12.5′S. 153°16.1′E.). They were housed in 60 L indoor raceways, supplied with flow-through seawater maintained at a constant temperature (25°C) under a 12 hour photoperiod. They were fed a diet of fresh *Sargassum* spp. and *Ecklonia radiata*. Under these conditions, *T. gratilla* broodstock could be reliably induced to spawn every 4–6 weeks.

Three macroalgae species were used in experiments; *Sargassum linearifolium* (Turner) C. Agardh, *Ulva* sp., and a geniculate coralline algae, *Corallina officinalis* Linnaeus. Algae were identified using standard texts [39], [40]. Macroalgae were collected in shallow subtidal waters at Charlesworth Bay, Coffs Harbour.

### Larval Production

Larval culture methodology was adapted from Dworjanyn and Pirozzi [11]. Adult *T. gratilla* (3–5 of each sex) were induced to spawn via intracoelomic injection of 1–2 mL of 1.0 M KCl. Eggs were pooled, pooled sperm was added incrementally and fertilisation was checked microscopically. When >95% of eggs had been fertilised, as evidenced by a fertilisation envelope, excess sperm was removed by gently washing the embryos in filtered seawater (filtered to 1.0 µm, and UV sterilised: hereafter referred to as FSW). Embryos were added at a concentration of 5–10 embryos.mL^−1^ to a 300 L cylindro-conical culture tank containing FSW that was gently aerated and maintained at 25°C. Larvae were fed from 3 days post-fertilisation with *Proteomonas sulcata* (Australian National Algae Culture Collection, strain CRFI01) at a rate of 5000 cells.mL^−1^. This was incrementally increased to 20–40,000 cells.mL^−1^ prior to settlement induction of the larvae. FSW was changed daily and culture tanks were exchanged weekly. Larval density fell from the initial 5–10 larvae.mL^−1^ to 1–4 larvae.mL^−1^ by 45 days post-fertilisation. Most losses occurred during tank exchanges. Larvae were classed as competent if pedicellaria and/or tube feet were observed (Mos, unpublished manuscript). Competent larvae began to appear in cultures from 19 days post-fertilisation.

### Effects of Natural Biofilm Plates as a Settlement Cue

To document settlement rates and post-settlement survival of *T. gratilla* when using natural biofilm settlement plates in a commercial scale trial, five replicate rectangular tubs (645×413×397 H mm), each holding 60 L, were assembled within a raceway and supplied with flow-through seawater (filtered 10 µm) at a rate of 1.6 L.min^−1^. The water level in the raceway was maintained at a depth of 200 mm to act as a water bath to maintain stable water temperatures within the tubs.

Biofilms were cultured in separate raceways for a minimum of seven weeks prior to use in experiments. As growth of the biofilms used in the first assay was slow, they were pulse fertilised with Yates® Thrive® Soluble All Purpose Plant Fertiliser (N 27%, P 5.5%, K 9.0%) at a rate of 0.02 g.L^−1^ at six weeks of age. Fertilisation was not required for the culture of biofilms used in later experiments. Biofilms were cultured on the following apparatus; a horizontal plastic base, four 350×450 mm vertical corrugated plastic plates and three 100×100 mm plates positioned between the larger plates, used as sub-samples of the large plates to minimise handling mortalities. One apparatus was positioned in each replicate tub one day prior to the start of the experiment. The biofilms cultured in this manner contained a mix of naturally-occurring bacteria, diatoms, algae and multi-cellular animals and are referred to as natural biofilms hereafter.

Larvae (∼50% competent, n = 250) were seeded twice into each settlement tub six days apart, with 40,000 and 30,000 larvae per replicate in each respective introduction. Water flow was stopped until swimming larvae were no longer observed (72 hrs), but gentle aeration was provided during this time and throughout the experiment. Temperature and salinity readings were recorded daily.

Settlement rates were visually assessed once swimming larvae were no longer observed, 72 hrs after the second larval introduction. Settled juveniles (approx. 0.5 mm diameter) were easily seen amongst the biofilm. All settled sea urchins were counted on the sides of the tubs, and within five haphazardly selected 50×50 mm quadrats on the bottoms of the tubs. Sea urchins were also counted on the three 100×100 mm plates in each of the settlement tubs to get an assessment of the number of urchins settled to the plates. These results were then extrapolated to give an estimate of the total number of settled urchins on each of these areas and within the replicate. Post-settlement survival was assessed weekly, using the same methods. As post-settlement mortality increased, larger sample sizes were used for the bottom of the tub and the plates. By the fourth week, the whole of the bottom of each tub, and all of the plates in each tub were surveyed for juvenile sea urchins.

### Effect of Temperature on Settlement and Post-Settlement Survival

To test the effect of temperature on settlement and early post-settlement survival of *T. gratilla*, larvae were induced to settle at five temperatures in regular increments from the ambient seawater temperature of 23°C to a maximum of approximately 33°C. Flow-through FSW was supplied to 60 L header tanks heated using thermostat controlled aquarium heaters. This seawater was supplied to ten replicate containers per treatment at a rate of 180 mL.hr^−1^. Each replicate container consisted of a 100 mL plastic jar that had a window covered in 250 µm mesh that maintained the water level at 30 mL. Competent larvae (n = 20 per replicate) were added to containers and induced to settle using *C. officinalis* (15 mm^2^) as a cue [11].

Settlement was assessed at 72 hours, after which settled juveniles were gently pipetted into new containers of the same design inoculated with the diatom *Nitzschia closterium* (Australian National Algae Culture Collection, CS-5). Juvenile sea urchins were maintained in these containers under a photoperiod of 16∶8 hours (light: dark) at the five temperatures for a further three weeks to assess post-settlement survival during the formation of the digestive tract [41]–[44]. *N. closterium* was added daily from liquid cultures to form a benthic layer available as food. Survival was assessed weekly, and temperatures were monitored twice daily.

### Settlement Assay Protocol

Settlement assays were conducted at 25°C in a temperature controlled laboratory with a photoperiod of 16∶8 hours (light: dark). For each treatment, ten replicate sterile plastic petri dishes (Sarstedt, Australia), with a diameter of 36 mm, were filled with 4.0 mL of autoclaved filtered seawater (hereafter referred to as ASW). 10–50 competent larvae were added per replicate. Ten replicates of a control treatment of ASW only were used in all settlement assays. Settlement was counted after 48 hours. Larvae were classed as settled if they were attached to a surface via tube feet and displayed eversion of the rudiment, all other larvae were classified as non-settled. The number of settled juveniles that possessed spines was also recorded. Results were expressed as a percentage of the total number of larvae added to the petri dish.

### Effects of Macroalgae Cues on Settlement

A settlement assay was conducted to compare the effectiveness of macroalgae, seawater conditioned with macroalgae, a mono-specific diatom and natural biofilm, and some combinations of these, on the induction of settlement of *T. gratilla*. Macroalgae and their extracts have been shown to induce varying rates of settlement of *T. gratilla* [11], [20]. A positive control treatment comprised of natural biofilm was collected using a glass slide to scrape 20 mm^2^ areas from the vertical plastic plates used to culture the natural biofilm. The diatom, *N. closterium*, was cultured using aseptic technique. 2 mL of culture (16.7±4.1×10^6^ cells.mL^−1^) was used to inoculate replicates (containing 2 mL of ASW) prior to the introduction of larvae. Pieces, approximately 15 mm^2^, of the macroalgae *S. linearifolium*, *Ulva* sp. and *C. officinalis* were used in respective treatments. Macroalgae-conditioned seawater was produced by adding 1 g wet weight of *S. linearifolium* or 0.5 g *C. officinalis* to 100 mL of FSW and aerating for 24 hours prior to commencement of the experiment. 2 mL of the respective conditioned seawater was used in each replicate (containing 2 mL of ASW). Combinations of *S. linearifolium* and *C. officinalis* conditioned seawater with natural biofilm or *N. closterium* were tested to determine if these additions could increase settlement rates in these treatments.

### Effects of Bacterial Cues on Settlement

Mixed consortia of bacteria were isolated from sources known to induce settlement of *T. gratilla*; the seaweeds *C. officinalis* and *S. linearifolium*, adult *T. gratilla* faeces and gut contents, larval culture tank deposits and a natural biofilm. Bacterial inoculants from these sources were obtained under aseptic conditions in a laminar-flow cupboard by the addition of approximately 10 g wet weight or 100 mm^2^ surface area of the respective sources of bacteria to 100 mL of ASW, which was shaken vigorously for 5 minutes. 10 mL of the resulting liquid was filtered (filter paper, Whatman® Grade 1) to remove large solids, and 100 µL of the filtrate was used to inoculate culture media. Bacteria were cultured in 100 mL of 1∶10 concentration of marine broth media (BD Difco™ Marine Broth 2216) in ASW for 48 hours prior to use in settlement assays. Each replicate petri dish was inoculated with 100 µL of the respective bacterial cultures. In pilot experiments, the volume of bacterial culture (1 mL, 100 µL, 10 µL) added to each replicate did not affect settlement (PERMANOVA not shown, F_2, 6_ = 1.416, P = 0.223). A commercial probiotic (Sanolife® by INVE Aquaculture), containing a mixed consortium of *Bacillus* spp., was cultured as per instructions, and used in the same way.

In parallel, six bacterial strains isolated from *C. officinalis* and *Amphiroa anceps*, (A2, A358, H33, C11, C38, C312; University of New South Wales bacterial strain collection) were cultured as for the mixed consortiums. Strains were selected as they had induced varying settlement rates for *Heliocidaris erythrogramma* [13] and other sea urchin larvae (Nielsen, unpublished data). To test if supernatants could induce settlement in the absence of bacteria, supernatants of each of the bacterial strains and the mixed consortiums were filtered (0.22 µm Millex® Millipore GP syringe filter) and 100 µL added to each replicate. Positive control treatments of *C. officinalis*, *S. linearifolium* and natural biofilm were prepared as previously described, along with equivalent proportions of adult *T. gratilla* faeces and gut contents. Larval culture tank deposits were prepared as for the natural biofilm. A control treatment of the bacterial culture medium was used, with replicates receiving 100 µL each.

### Effects of Bacterial Culture Medium, Age and Presence of Macroalgae on Settlement

The effect of culture medium was examined using surface and liquid media at varying concentrations of marine broth (0.1, 1 and 10% marine broth concentration in ASW). Liquid media were created as previously described. Surface media were created by the addition of 1.5% agar (Becton Dickinson, Bacto™ Agar 214030) to the various concentrations of marine broth in ASW. 1 mL of the respective media was added to ten replicate sterile plastic petri dishes (36 mm, Sarstedt, Australia). Surface media were inoculated by the addition of 100 µL of bacterial inoculant, derived from *C. officinalis* as previously described. The inoculants were spread across the surface of the media and bacteria were left to attach for 30 minutes before the media was covered by 4 mL of ASW. Cultures were left for 48 hours before settlement assays were conducted. ASW was replaced with fresh ASW just prior to the introduction of larvae. Controls of *C. officinalis*, natural biofilm, agar, surface and liquid media were used respectively.

To assess the effectiveness of mixed consortia of bacteria to induce settlement of *T. gratilla* as a function of age, bacterial cultures aged for 24, 48, 72 and 96 hours were used in a settlement assay. A standard liquid culture was created using bacteria isolated from *C. officinalis* as previously described. This standard culture was used to inoculate ten replicates of the (10% marine broth in ASW) surface media every 24 hours for a period of 72 hours. The final surface cultures were prepared 24 hours prior to commencement of the settlement assay. Controls of *C. officinalis*, natural biofilm, agar and surface media were used.

To test whether there was a cofactor produced by *S. linearifolium* that influenced the capacity of bacteria to induce settlement bacteria were assayed in the presence/absence of *S. linearifolium*. Bacteria were isolated from *S. linearifolium* by scraping thalli three times per side across semi-solid agar (5% in FSW). Thalli of *S. linearifolium* were cleaned by scraping across semi-solid agar, and the combination of scraping and steeping in iodine (10% in FSW). These treatments are known to effectively remove almost all bacteria from the treated macroalgae [11]. An assay was done using the agar containing bacteria scraped from *S. linearifolium* alone and in combination with cleaned *S. linearifolium* thalli. Cleaned and uncleaned thalli of *S. linearifolium* alone, and agar without bacteria were also assayed as controls.

### Trial of a Highly Inductive Settlement Cue

A combination of *S. linearifolium* conditioned seawater and natural biofilm was shown to induce significantly more settlement than natural biofilm alone in the laboratory assays (see Results). To test whether this combination works on a commercial scale, a trial was conducted to compare the combination of natural biofilm and *S. linearifolium* conditioned seawater (60 L per replicate, 50% conditioned seawater in FSW) to natural biofilm in FSW only. The experimental apparatus used was as previously described, except three large plates were used instead of four. There were five replicates for each treatment, with 32,500 larvae (approx. 65% competent, n = 250) added per replicate. Settlement rates were assessed once there were no swimming larvae observed in the water column (96 hrs) and water flow was started at 1.6 L.min^−1^. Settlement rates and post-settlement survival were assessed as previously described.

### Statistical Analysis

Data were analysed using permutational analysis of variance (PERMANOVA [45]). PERMANOVA compares the F statistics to a distribution generated by multiple random permutations of the analysed data, thus liberating it from the formal assumptions of traditional ANOVA [45]. Settlement assays often contain heterogeneous variances among treatments, typically due to no settlement in controls. PERMANOVA avoids the use of data transformations and the exclusion of controls during analysis. Pair-wise comparisons of untransformed data were generated using Euclidean distance, utilising approximately 9999 permutations of the raw data. A Monte Carlo procedure was used when the number of unique permutations was low. Repeated measures analysis of survival was conducted for the temperature and large scale macroalgae-conditioned seawater trials. Pair-wise post-hoc tests were performed if PERMANOVA results indicated that there were significant differences between treatments. *A priori* planned contrasts were conducted between supernatant and bacteria, and surface and liquid media treatments respectively. Analyses were conducted using Primer 6 (Primer-E, Plymouth) with PERMANOVA^+^ extension (v.6.1.7) software.

## Results

### Effects of Natural Biofilm Plates as a Settlement Cue

**Figure 1:**
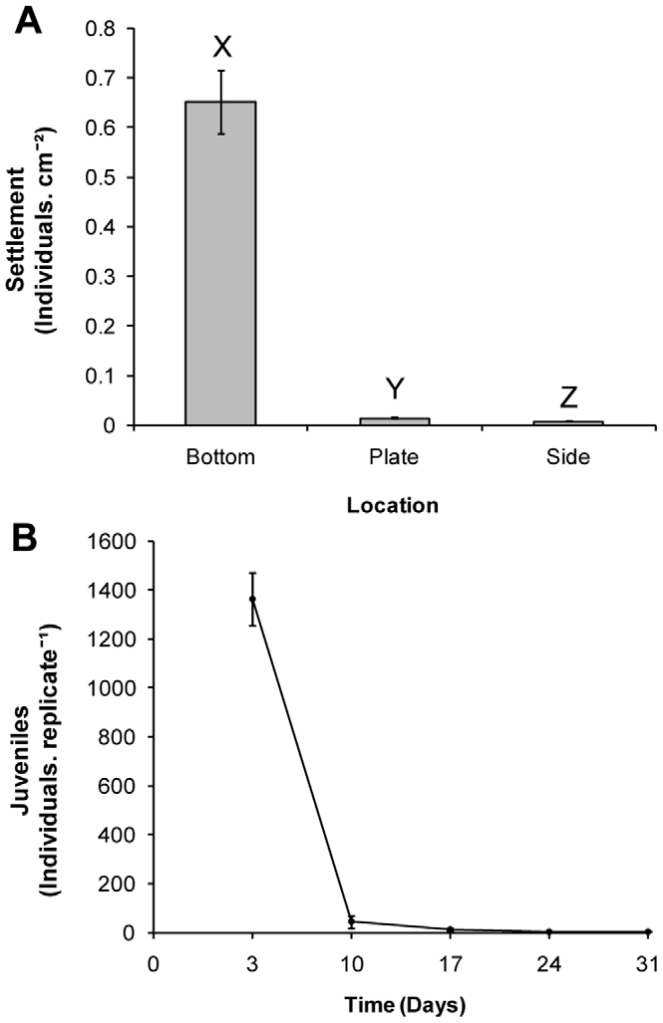
Settlement and post-settlement survival of *Tripneustes gratilla* induced to settle using natural biofilm in a commercial scale trial. (A) Location of newly settled sea urchins at first census. Letters denote statistical differences (PERMANOVA, post-hoc pair-wise test). (B) Survival to 31 days. Data are means ± SE.

Settlement of *T. gratilla* occurred in all replicates, but was low with a mean settlement rate of 1.94% (±0.16 SE). Larvae did not settle at equal rates to different areas of the tubs (Fig. 1A, PERMANOVA, F_2, 12_ = 101.57, P = 0.0001); the highest settlement occurred on the bottom of the tubs, with significantly less settlement recorded on vertical surfaces (post-hoc pair-wise test, P<0.05). Post-settlement survival was poor, <1% of settled juveniles were alive within three weeks (Fig. 1B). Environmental conditions were stable for the first ten days of the experiment (salinity 35.0 ppt, water temp. 22.2±1.0 SD°C) but rainfall after this time caused salinity to fluctuate (33.9–35.0 ppt) and the water temperature ranged from 19.2–23.2°C.

### Effect of Temperature on Settlement and Post-settlement Survival

**Figure 2:**
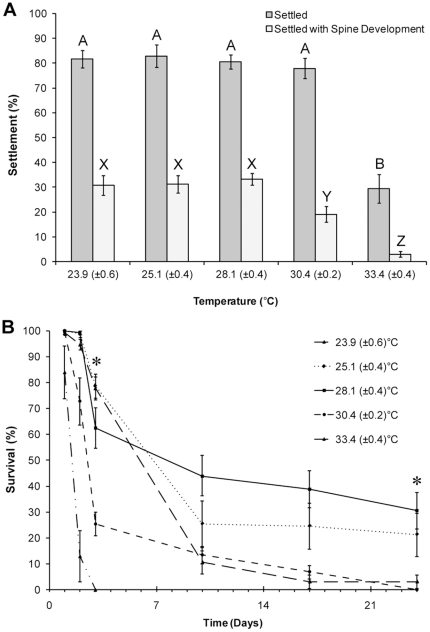
The effect of temperature on settlement and post-settlement survival of *Tripneustes gratilla* in a small scale laboratory trial. (A) Settlement and post-settlement spine development. Letters denote significant differences (PERMANOVA, post-hoc pair-wise test). (B) Survival to 24 days. * denotes significant difference between treatments (PERMANOVA, post-hoc pair-wise test). Data are means ± SE.

The mean temperatures (±S.D.) of the five treatments used were 23.9 (±0.6), 25.1(±0.4), 28.1 (±0.4), 30.5 (±0.2) and 33.4 (±0.4)°C respectively. Temperature significantly affected settlement, with less settlement at the highest temperature (Fig. 2A, PERMANOVA, F_4, 45_ = 29.283, P = 0.0001, followed by post-hoc pair-wise test, P<0.05). Development of spines in newly settled sea urchins was also affected by temperature (Fig. 2A, F_4, 45_ = 17.722, P = 0.0001); temperatures above 30°C significantly reduced the percentage of urchins that developed spines post-settlement (post-hoc pair-wise test, P<0.05).

Post-settlement survival was significantly affected by temperature. Survival of settled urchins was statistically similar in all treatments after 24 hours (Table 1; followed by post-hoc pair-wise test, Fig. 2B, not presented), however there was a decrease in survival with each increase in temperature over 28°C at 3 days (Table 1; followed by post-hoc pair-wise test, Fig. 2B, P<0.05). At 24 days, there was significantly lower survival in the lowest temperature treatment and in the temperature treatments above 30°C (Table 1; followed by post-hoc pair-wise test, Fig. 2B). Material was observed within the digestive tract of the urchins by ten days regardless of temperature treatment, evidence that urchins had begun feeding by that time.

**Table 1:** Repeated measures PERMANOVA examining survival of *Tripneustes gratilla* at 6 time points to 24 days at five different temperatures.

Source	df	MS	*F*	*P*	Unique Permutations
Treatment	4	20896	73.334	**0.0001**	9952
Time	5	63729	233.66	**0.0001**	9949
Treatment × Time	20	2542.3	8.9223	**0.0001**	9906
Residual	270	284.94			
Total	299				

Significant differences (P<0.05) are in bold; df, degrees of freedom, MS, mean square.

### Effects of Macroalgae Cues on Settlement

**Figure 3:**
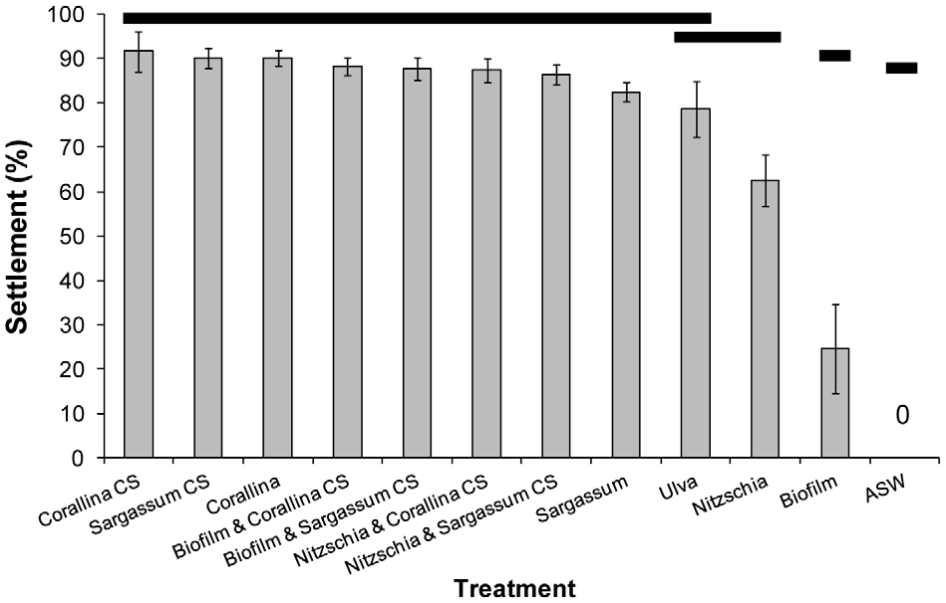
The settlement of *Tripneustes gratilla* to macroalgae, macroalgae-conditioned seawater, a mono-specific diatom and natural biofilm. The full species names are presented in the text. CS = Conditioned Seawater, ASW = Autoclaved Seawater. Data are means ± SE. Bars denote statistical differences (PERMANOVA, post-hoc pair-wise test).

*T. gratilla* settled in response to all treatments, except the ASW control (Fig. 3). Settlement to macroalgae and their respective conditioned seawater was not significantly different (Fig. 3, PERMANOVA, F_11, 108_ = 51.367, P = 0.0001, followed by post-hoc pair-wise test, P<0.05). Natural biofilm and *N. closterium* induced significantly lower settlement than macroalgae and conditioned seawater treatments, but settlement to these treatments was significantly greater when they were combined with macroalgae-conditioned seawater.

### Effects of Bacterial Cues on Settlement

**Figure 4:**
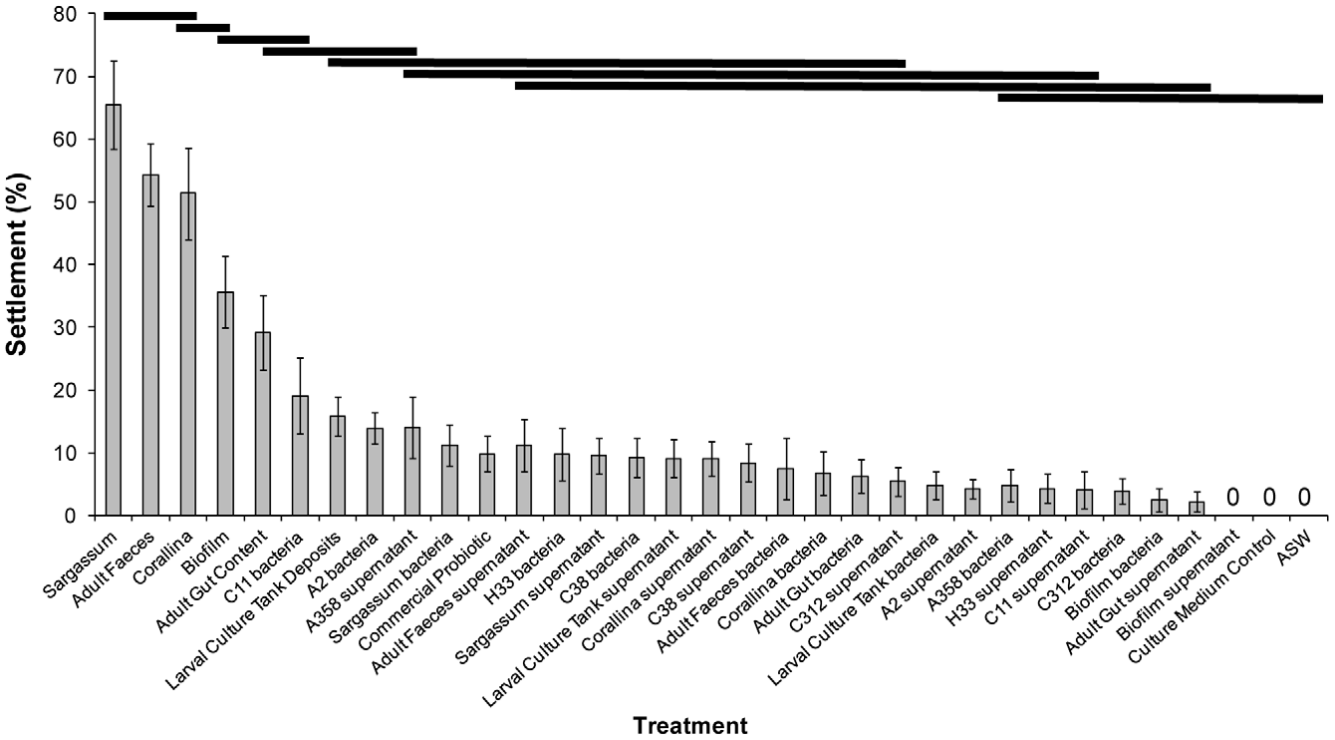
The settlement of *Tripneustes gratilla* to mixed consortia, single strains and supernatants of bacteria isolated from macroalgae, biofilms and adult conspecifics. The full species names are presented in the text. ASW = Autoclaved Seawater. Data are means ± SE. Bars denote statistical differences (PERMANOVA, post-hoc pair-wise test).

All treatments, except the natural biofilm derived bacterial supernatant, ASW and marine broth control, induced settlement of *T. gratilla* (Fig. 4). There were significant differences between treatments, which formed a hierarchy of groupings with overlapping significance (Fig. 4, PERMANOVA, F_32, 297_ = 18.566, P = 0.0001, followed by post-hoc pair-wise test, P<0.05). All mixed consortia of bacteria induced significantly lower settlement than the source they were isolated from. All mixed consortia, single strains and supernatants induced significantly lower settlement than adult faeces and macroalgae positive controls. Bacterial treatments, in particular the C11 strain, did not significantly differ from natural biofilm, adult gut contents or larval culture tank deposits. There were no significant differences between settlement to mixed consortiums and single strains, or between bacteria and their respective supernatant, except for the C11 and A2 strains. An *a priori* planned contrast revealed no significant difference in the settlement to bacteria and supernatants (Fig. 4, F_1, 238_ = 1.2341, P = 0.2701).

### Effects of Bacterial Culture Medium, Age and Presence of Macroalgae on Settlement

**Figure 5:**
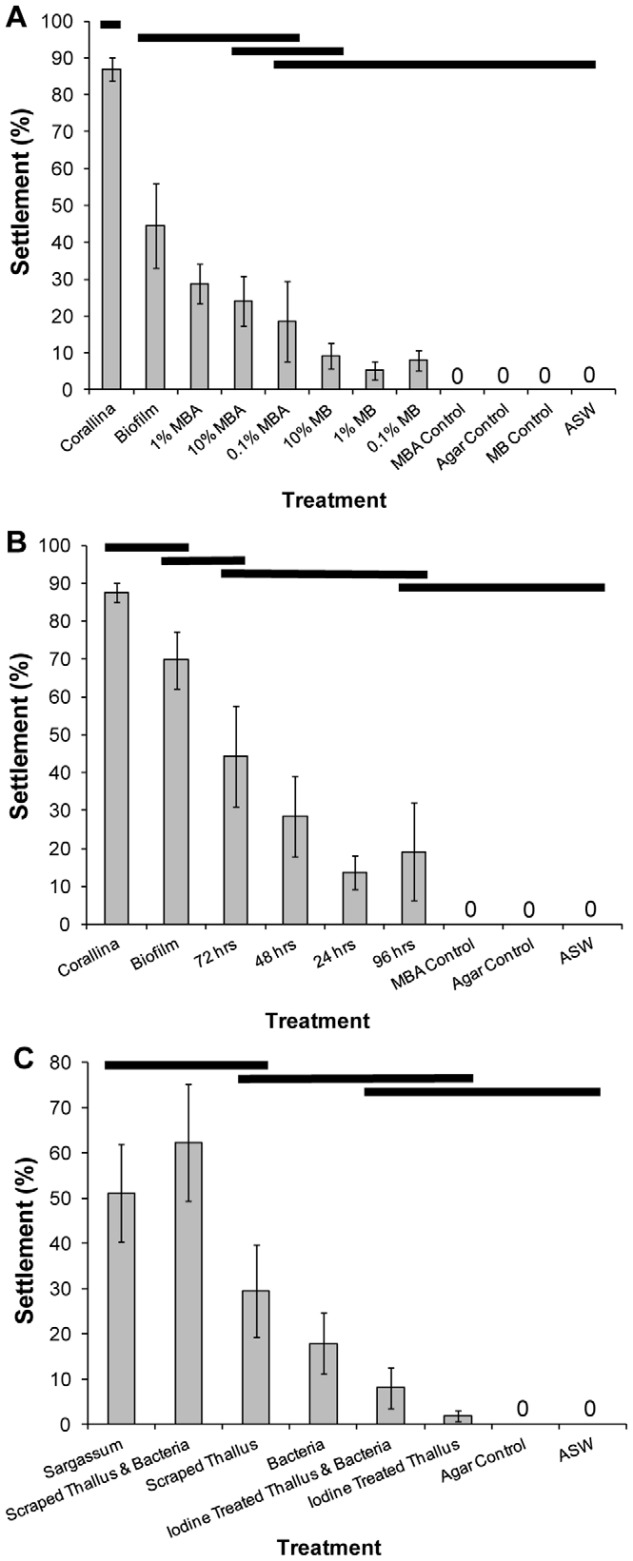
The effect of (A) culture medium, (B) culture age and (C) presence of macroalgae on induction of settlement of *Tripneustes gratilla* by mixed consortiums of bacteria. The full species names are presented in the text. MBA = Marine Broth/Agar surface medium, MB = Marine Broth in ASW liquid medium, ASW = Autoclaved Seawater. X-axis percentages denote concentration of Marine Broth in the culture medium. Bars denote statistical differences between treatments (PERMANOVA, post-hoc pair-wise test). Data are means ± SE.

Settlement of *T. gratilla* occurred to all treatments, except the ASW, agar and culture medium controls which did not induce settlement (Fig. 5A, B, C). There were significant differences in settlement to mixed consortia of bacteria grown in different culture media (Fig. 5A, PERMANOVA, F_11, 78_ = 23.002, P = 0.0001). Treatments formed a hierarchy of groupings with overlapping significance (Fig. 5A, post-hoc pair-wise test, P<0.05). An *a priori* planned contrast revealed significant difference in the settlement to surface and liquid media (Fig. 5A, F_1, 58_ = 18.101, P = 0.0004, not presented), with higher settlement to bacteria cultured on surface media. Bacterial treatments induced significantly less settlement than the *C. officinalis* control, regardless of the type of culture medium. There was no obvious effect of concentration of nutrients in media on settlement rate in either the liquid or solid media. Settlement to mixed consortia of bacteria of different culture ages did not significantly differ (Fig. 5B, F_8, 36_ = 17.205, P = 0.0001), but significantly differed from the *C. officinalis* and natural biofilm controls, except for the natural biofilm and 72 hours treatments (post-hoc pair-wise test, P<0.05).

Bacteria removed from *S. linearifolium* induced significantly lower settlement than the *S. linearifolium* control (Fig. 5C, F_7, 31_ = 10.542, P = 0.0001, followed by post-hoc pair-wise test, P<0.05). The combination of bacteria and scrape-iodine treated thalli did not significantly increase settlement compared to the bacteria only treatment. Bacteria induced higher settlement when combined with the thallus they were removed from, although this was not significantly different than the *S. linearifolium* control.

### Trial of a Highly Inductive Settlement Cue

**Figure 6:**
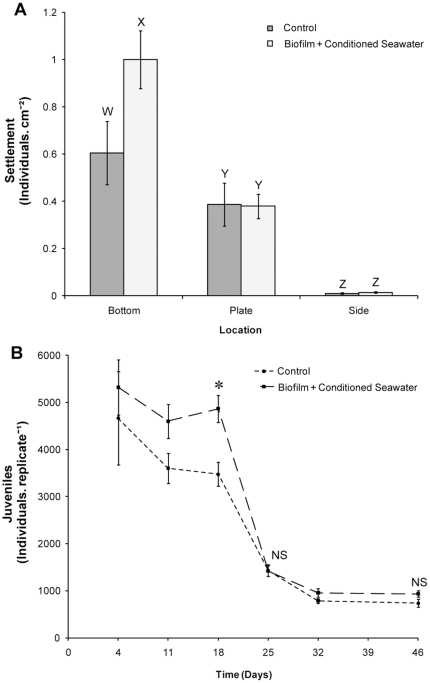
The effect of *Sargassum linearifolium* conditioned seawater in combination with natural biofilm settlement plates on settlement and post-settlement survival of *Tripneustes gratilla* in a commercial scale trial. (A) Location of settlement. Letters denote statistical differences (PERMANOVA, post-hoc pair-wise test). (B) Survival to 46 days. Control = natural biofilm plates, Biofilm + Conditioned Seawater = natural biofilm plates and *S. linearifolium* conditioned seawater. * denotes significant difference between treatments (PERMANOVA, P<0.05), NS = not significant. Data are means ± SE.

Settlement of *T. gratilla* occurred in both the control and *S. linearifolium* conditioned seawater treatments, with mean settlement rates of 14.4% (±3.1 SE) and 16.4% (±1.8 SE) respectively. There was significantly higher settlement to the bottom of tubs in the *S. linearifolium* conditioned seawater treatment than in the natural biofilm control (Table 2; followed by post-hoc pair-wise test, P<0.05, Fig. 6A). Regardless of treatment, the highest settlement occurred on the bottom of the tubs, with significantly less settlement recorded on vertical surfaces (Table 2; followed by post-hoc pair-wise tests, Fig. 6A).

**Table 2:** PERMANOVA analysis of settlement of *Tripneustes gratilla* to *Sargassum linearifolium* conditioned seawater after 96 hours.

Source	df	MS	*F*	*P*	Unique Permutations
Treatment	1	0.12906	3.9495	0.0609	9865
Settlement Position	2	1.613	49.362	**0.0001**	9961
Treatment × Settlement Position	2	0.13075	4.0014	**0.0278**	9953
Residual	24	0.032676			
Total	29				

Significant differences (P<0.05) are in bold; df, degrees of freedom, MS, mean square.

The mean number of juveniles per replicate remained significantly different between treatments at 18 days, but was not significant at 25 days (Table 3; followed by post-hoc pair-wise test, Fig. 6B). This shift in significance corresponded with a period of high mortality (Fig. 6B). Mortality did not appear to correspond with the availability of food as natural biofilm was present throughout the experiment, however this was not quantified.

**Table 3:** PERMANOVA repeated measures analysis examining survival of *Tripneustes gratilla* at day 18, 25 and 46 after settlement to natural biofilm plates and *Sargassum linearifolium* conditioned seawater.

Source	df	MS	*F*	*P*	Unique Permutations
Treatment	1	2.0806×10^6^	13.439	**0.0016**	9838
Time	2	3.1743×10^7^	205.04	**0.0001**	9955
Treatment × Time	2	1.4361×10^6^	9.2763	**0.0023**	9957
Residual	24	1.5482×10^5^			
Total	29				

Significant differences (P<0.05) are in bold; df, degrees of freedom, MS, mean square.

The trial was ended after 46 days, corresponding approximately to when the bulk of sea urchins were capable of transitioning to a seaweed diet. There was no significant difference between treatments in the mean number of juveniles per replicate at this time (Table 3; followed by post-hoc pair-wise test, Fig. 6B). Survival rates of settled juveniles to 46 days for the natural biofilm control and *S. linearifolium* conditioned seawater treatment were 18.47% (±3.54 SE) and 18.51% (±2.81 SE) respectively. Environmental conditions remained consistent throughout the experiment. Salinity was 35.0 ppt and mean temperatures were 24.4°C (±1.3 SD).

## Discussion

A critical phase in sea urchin aquaculture is the transition from swimming larvae to the onset of post-settlement feeding. This phase accounts for the largest losses in closed-cycle rearing of sea urchins, associated with highly variable settlement and poor post-settlement survival [7], [8]. This was clearly demonstrated in this study as settlement rates were less than 2% and post-settlement survival was less than 1% of settled individuals when the current commercial methodology was tested. Temperature was an important factor in determining settlement rate and post-settlement development and survival. The type of settlement cue larvae were exposed to was an important determinant of settlement success; in particular the macroalgae *S. linearifolium* and *C. officinalis*, and seawater conditioned with these macroalgae, were highly effective at inducing settlement. Bacteria isolated from these and other natural sources induced significant settlement of larvae but not at rates equivalent to the sources from which they were isolated. Attempts to increase induction of settlement by mixed consortia and individual strains of bacteria, using different culture techniques, culture times, and nutrient concentrations did not lead to any large improvements in settlement. Whilst it was found that settlement could be enhanced in a large scale trial by introducing a dissolved algal cue to natural biofilm settlement plates, high post-settlement mortality meant these gains were lost after only a few weeks.

In this study, *T. gratilla* did not settle in the absence of a cue, supporting evidence that settlement cues are obligatory [11] (but see [20]). *T. gratilla* settled in response to a wide variety of cues, including natural biofilms, adult conspecifics, macroalgae, a mono-specific diatom, bacteria and conditioned seawater, similar to the wide response to cues demonstrated previously for this [11] and other sea urchin species [13], [14]. The macroalgae cues (*C. officinalis* and *S. linearifolium*) that consistently induced high settlement here also correspond with the most effective cues for inducing settlement in other studies [11], [20]. *T. gratilla* also settled in response to six single strains and multiple mixed consortia of bacteria, and their supernatants, which were isolated from macroalgae and other sources that induce settlement. Single strains or species of bacteria have been to shown to induce settlement in a range of marine invertebrates [46], but prior to this study, only one species of sea urchin (*Heliocidaris erythrogramma*) has been shown to settle in response to single strains of bacteria isolated from macroalgae [13]. However, in contrast to the results obtained by Huggett et al. [13], settlement rates to the cultured bacteria here were always significantly lower than the sources from which bacteria were isolated. One possible explanation for this is that the difference in bacterial culture methods between the studies may have resulted in bacteria of differing physiological states [47].

The physiological state of bacteria can determine the metabolites that they produce [38], possibly affecting their capacity to induce settlement of marine invertebrate larvae. The age of bacterial cultures can affect settlement [48], but in this study there was no difference in settlement to mixed consortia of bacteria cultured for 24, 48, 72 and 96 hours. There was evidence that bacteria cultured on a surface induced more settlement than bacteria from liquid cultures, similar to other studies on marine invertebrates [49]–[51]. It has been suggested macroalgae may produce a cofactor that enhances settlement to bacterial biofilms [11], [52], but no evidence of this was found here when macroalgae and their bacterial biofilms were partitioned. Macroalgae may therefore simply provide a good surface for the growth of bacterial biofilms, allowing a physiological state that produces inductive compounds [50], [52]. It remains unclear then as to why the bacterial cultures here induced less settlement than macroalgae controls.

Settlement was affected by temperature when larvae were exposed to extremes above normal habitat parameters [36], and post-settlement development and survival was optimal between 24–28°C. Therefore this temperature range is recommended as the optimal temperature range for settlement and early post-settlement culture of *T. gratilla*, corresponding to the optimal temperature ranges for larval culture [53], [54] and juvenile somatic growth (Mos, unpublished data). This is the first time that the effect of temperature on settlement has been tested for any sea urchin but supports evidence that temperature is an important determinant of settlement and post-settlement survival for marine invertebrates [55]–[57]. Lower temperatures than those tested here are likely to reduce settlement rates given the tropical/subtropical distribution of *T. gratilla*.

The manipulation of settlement cues and temperature demonstrate the potential of these factors to optimise settlement, however the fate of settled larvae should be followed in commercial scale trials to determine if highly inductive settlement cues bolster efficiency in the long term. This study found that settlement rates could be significantly bolstered on a commercial scale by the addition of *S. Linearifolium* conditioned seawater to natural biofilm settlement plates, but these gains were quickly lost as high post-settlement mortality resulted in no difference in survival between treatments by 25 days. Similarly high mortalities occurred during this period in the natural biofilm and temperature trials (Fig. 1B, 2B). Grosjean et al. [7] recorded that the highest post-settlement mortalities for the sea urchin *Paracentrotus lividus* cultured on a commercial scale occurred ‘probably during the first few weeks’. This trend is not limited to sea urchins, with the highest post-settlement mortalities for abalone occurring within the first few weeks [29], [33], [58], [59]. The periods during which highest mortalities were observed here correspond to the non-feeding period of post-settlement development of the digestive system in echinoids [41]–[44]. The energy and materials required for post-settlement development of sea urchins is supplied by disassociated larval structures [60] and energy obtained during the larval stage from both maternal provisioning and planktotrophic feeding. Byrne et al. [39], [61] demonstrated that the early post-settlement performance of *T. gratilla* was influenced by triglyceride stores. Similarly, larval nutrition has been shown to have an important role in post-settlement development and survival for several other sea urchins [42], [62]–[64].

The nutritional status of larvae is currently not assessed in determining the competence of sea urchin larvae to settle. Competence is generally determined only by the ability of larvae to respond to settlement cues. This may be flawed given that there is evidence that more developed larvae store greater amounts of energy [41] and larvae may develop the ability to detect and respond to cues prior to completing larval development [65]. For example, the presence and size of the larval rudiment is often used to determine competence of *T. gratilla* [11], [20], however the larval rudiment is generally not the developmental endpoint of planktotrophic sea urchin larvae, as pedicellaria, tube feet and spines develop afterwards [66]–[68]. This points to the need to further investigate the roles of competency and maternal and larval nutrition in determining the early post-settlement survival of sea urchins. This may lead to methods which further increase post-settlement survival, thus removing the biggest bottleneck to efficient sea urchin aquaculture.
